# Molecular Recognition of Surface *Trans*-Sialidases in Extracellular Vesicles of the Parasite *Trypanosoma cruzi* Using Atomic Force Microscopy (AFM)

**DOI:** 10.3390/ijms23137193

**Published:** 2022-06-28

**Authors:** Alexa Prescilla-Ledezma, Fátima Linares, Mariano Ortega-Muñoz, Lissette Retana Moreira, Ana Belén Jódar-Reyes, Fernando Hernandez-Mateo, Francisco Santoyo-Gonzalez, Antonio Osuna

**Affiliations:** 1 Grupo de Bioquímica y Parasitología Molecular (CTS 183), Departamento de Parasitología, Campus de Fuentenueva, Instituto de Biotecnología, Universidad de Granada, 18071 Granada, Spain; lissette.retanamoreira@ucr.ac.cr; 2 Departamento de Microbiología Humana, Facultad de Medicina, Universidad de Panamá, Ciudad de Panamá 0824, Panama; 3 Unidad de Microscopía de Fuerza Atómica, Centro de Instrumentación Científica, Universidad de Granada, 18071 Granada, Spain; flinaor@ugr.es (F.L.); mortegam@ugr.es (M.O.-M.); 4 Departamento de Parasitología, Facultad de Microbiología, Universidad de Costa Rica, San José 2060, Costa Rica; 5 Centro de Investigación en Enfermedades Tropicales (CIET), Universidad de Costa Rica, San José 2060, Costa Rica; 6 Grupo de Física de Fluidos y Biocoloides, (FQM 115), Excellence Research Unit Modeling Nature (MNat), Departamento de Física Aplicada, Facultad de Ciencias, Universidad de Granada, 18071 Granada, Spain; ajodar@ugr.es; 7 Departamento de Química Orgánica, Facultad de Ciencias, Universidad de Granada, 18071 Granada, Spain; fhmateo@ugr.es (F.H.-M.); fsantoyo@ugr.es (F.S.-G.)

**Keywords:** *Trypanosoma cruzi*, trypomastigote, extracellular vesicles, *trans*-sialidase, molecular recognition, atomic force microscopy

## Abstract

*Trans*-sialidases (TS) are important constitutive macromolecules of the secretome present on the surface of *Trypanosoma cruzi* (*T. cruzi*) that play a central role as a virulence factor in Chagas disease. These enzymes have been related to infectivity, escape from immune surveillance and pathogenesis exhibited by this protozoan parasite. In this work, atomic force microscopy (AFM)-based single molecule-force spectroscopy is implemented as a suitable technique for the detection and location of functional TS on the surface of extracellular vesicles (EVs) released by tissue-culture cell-derived trypomastigotes (Ex-TcT). For that purpose, AFM cantilevers with functionalized tips bearing the anti-TS monoclonal antibody mAb 39 as a sense biomolecule are engineered using a covalent chemical ligation based on vinyl sulfonate click chemistry; a reliable, simple and efficient methodology for the molecular recognition of TS using the antibody-antigen interaction. Measurements of the breakdown forces between anti-TS mAb 39 antibodies and EVs performed to elucidate adhesion and forces involved in the recognition events demonstrate that EVs isolated from tissue-culture cell-derived trypomastigotes of *T. cruzi* are enriched in TS. Additionally, a mapping of the TS binding sites with submicrometer-scale resolution is provided. This work represents the first AFM-based molecular recognition study of Ex-TcT using an antibody-tethered AFM probe.

## 1. Introduction

The flagellated protozoan parasite *Trypanosoma cruzi* (*T. cruzi*), etiologic agent of Chagas disease or American trypanosomiasis, displays a great variety of glycoconjugates on its surface [1]. In the mammalian trypomastigote infective stage of the life cycle of the parasite, these glycoconjugates include glycosylphosphatidylinositol (GPI)-anchored proteins such as the *O*-glycosylated mucin-associated surface proteins (MASPs) and multiple glycan-binding proteins belonging to the *trans*-sialidase (TS) family [2]. In particular, at least 140 different proteins -classified into different groups according to their structure, function and activity-constitute the TS multigene family. 

The expression of TS is particularly relevant because glycointeractions involving sialic acid (SA) are essential for infectivity, escape from immune surveillance and pathogenesis exhibited by *T. cruzi* [1,2,3,4,5]. Among the expressed molecules of the TS family, not all present TS activity, being accordingly referred as active (aTS) and inactive (iTS) members. It has been well-established that the known disability of *T. cruzi* for *de novo* synthesis of SA is overcomed by the use of aTS enzymes and their ability to modulate the sialoglycophenotype of both the parasite and host cell’s glycans, by cleaving terminal SA residues from the host donor´s glycoconjugates and transferring them to the terminal β-linked galactose residues of the mucins on the surface of the parasite. Additionally, the non-enzymatically active iTS display lectinic properties, although both types of TSs share the same substrate specificity for α-2,3-SA-containing glycotopes. During an infection with *T. cruzi*, aTS members act as virulence factors in mammals coursing the acute phase [5], while iTS play a pathogenic role modulating events related to adhesion and invasion of the parasite into the host cells. Since SA-containing glycotopes modulate the host immune system, the TS-mediated changes in the sialylation of parasite’s mucin-like molecules and host cell glycoconjugates perturb critical physiological events, including the induction of an effective immune response.

The controlled shedding of TS into the bloodstream has been elucidated as a key evolution strategy for the mentioned trypomastigote´s manipulation of the surface sialylation pattern. In fact, TS are one of the most significant constitutive macromolecules of the secretome of *T. cruzi* [6] where they are anchored on the membrane of extracellular vesicles (EVs) as associated GPI-anchored proteins [7,8]. The term EVs design the collection of diverse membrane-bound entities delimited by a lipid bilayer that are liberated into the extracellular space by prokaryotic and eukaryotic cells. According to the agreement proposed by the International Society for Extracellular Vesicles (ISEV), EVs are classified by their size, biogenesis and composition, being the more relevant subtypes (i) exosomes (Ex) (20–200 nm), (ii) ectosomes (200–1000 nm) and apoptotic blebs (>1000 nm) [9]. It has been unveiled that EVs are key elements of an efficient strategy for cell-to-cell communication in view of the delivery of their cargo to short or long distances between the cells, acting through EV uptake or receptor-mediated interactions [10]. Although EVs are produced in the different life stages of *T. cruzi*, EVs released in the trypomastigote stage of *T. cruzi* may favor parasite´s survival, evasion of complement-mediated lysis, and setting a regulatory immune response, allowing parasite´s persistence [11,12,13,14,15]. Proteomics has revealed the complex composition of EVs, showing that trypomastigote´s vesicles contain most of the parasite´s cell-surface proteins, and that these vesicles express more TS and show higher adhesion values than EVs secreted by the epimastigote stage of *T. cruzi*, the non-infective form for mammalian cells [16]. Studies to clarify the protein turnover of the membranes of trypomastigotes have evidenced that TS and its mucin targets are separately distributed on the surface and contained in different and highly stable membrane microdomains. This location results too far off for the trypomastigote´s surface-anchored TS to sialylate mucins, a role that is played by the shed TS integrated into EVs instead [4]. 

The actual knowledge concerning the membrane structure of the trypomastigote stage of *T. cruzi* and its EVs in a glycobiological perspective has been acquired by a multidisciplinary approach using advanced microscopy techniques and biochemical methods [1,16]. Among the nanoscopic technologies, atomic force microscopy (AFM) is a powerful high-resolution imaging and force sensing technology that has demonstrated its utility to elucidate the structural characterization of pathogenic protozoa [17,18] including *T. cruzi* [4,19,20], and sub-cellular structures such as EVs [21,22,23,24] and other biological specimens. In the case of EVs, AFM represents an attractive alternative for the determination of the morphology, structure and composition, and for the quantification of biophysical characteristics (stiffness, Young’s modulus, and adhesion force, work of adhesion, hysteresis, dissipation, and relaxation times). In particular, AFM-based single molecule-force spectroscopy is the only technique for detecting the molecular recognition forces between two molecules with subnanometric precision and high sensitivity of the order of nanometers and picometers [25,26,27,28]. This technique requires the functionalization of the tip of the cantilever with a molecule (ligand, receptor or antibody) that will approach until contact with a surface in which the molecule to which they bind and interact (receptor, ligand or antigen) is immobilized [29,30,31]. After the contact, the tip is retracted and the breaking points of the interaction forces between the two molecules are quantified through the force-distance curves obtained. The technique allows the nanomechanical mapping and imaging of the locations of binding sites, demonstrating the unquestionable capability of AFM to measure biologically specific rupture forces of molecular complexes. 

Considering the importance of the SA-containing glycans in *T. cruzi*-host cell interplay, the orchestrated role played by TS of EVs in the sialylation events and the interest in EVs for diagnostic and therapeutic applications, we have devoted previously efforts to elucidate different biophysical parameters of EVs of epismatogotes and trypomastigotes of *T. cruzi* using the AFM imaging and spectroscopy analysis (Young’s modulus, stiffness and adherence) [16]. To gain further insights into this forefront and intriguing topic related to the pathology and infectivity of *T. cruzi*, in this work we describe the implementation of AFM-based single molecule-force spectroscopy as a suitable technique for the surface mapping of TS in EVs released by tissue-culture cell-derived trypomastigotes (Ex-TcT) of *T. cruzi.* The distinctive and strong noncovalent interaction between TS and the anti-TS monoclonal antibody (mAb 39) motivated us to prepare and evaluate an AFM cantilever-based probe for the molecular recognition of TS of Ex-TcT. For that purpose, we developed an ad hoc tip functionalization methodology based on vinyl sulfonate (VSO) click-chemistry for the fabrication of a highly specific antibody-tethered AFM probe [32]. The data obtained demonstrate that the membranes of the EVs of trypomastigotes are enriched in TS. Besides, a mapping of the TS binding sites with submicrometer-scale resolution is also provided. 

## 2. Results

### 2.1. Preparation of Functionalized Cantilevers

**Figure 1:**
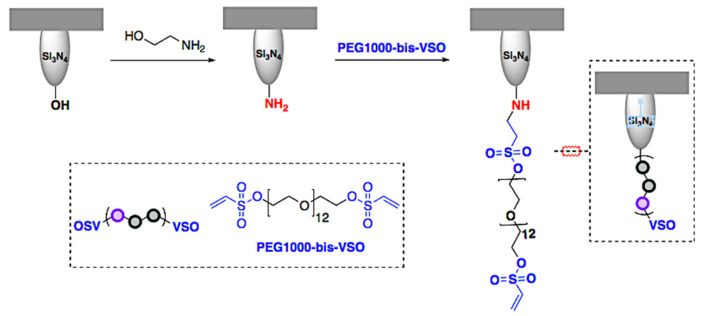
Preparation of the tip bounded to PEG1000-VSO.

**Figure 2:**
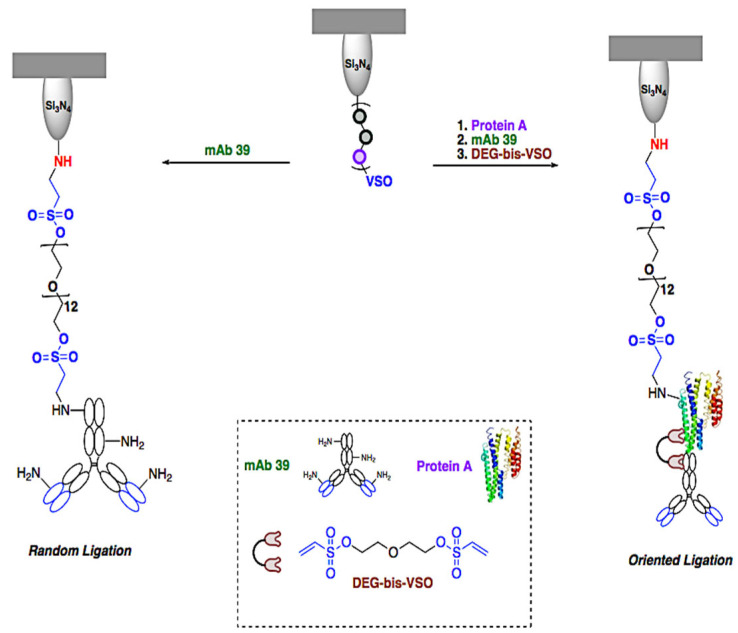
Random and oriented ligation of anti-TS mAb 39 antibody to PEG-VSO functionalized tips.

Figure 1 and Figure 2 summarize the preparation process of anti-TS mAb 39 functionalized AFM tips. The anti-TS mAb 39 antibody was linked to the AFM cantilever surface using a flexible poly (ethylene glycol) 1000 (PEG1000) chain as a molecular spacer. This is a well-established methodology that guarantees the maximization of the mobility of the anti-TS mAb 39 antibody while sensing the biomolecule and the efficiency in the recognition of the TS enzyme counterpart while minimizing nonspecific interactions with the EV surface [33]. To this end, the homobifunctional PEG1000-bis-vinyl sulfonate crosslinker (PEG1000-bis-VSO) was employed, prepared from commercial PEG1000 [32] for the incorporation of end-tethering units bearing pendent reactive VSO groups. A cantilever with a spring constant of 0.6 N/m was selected, to provide sensitivity in the order of pN in force. This range proved to be suitable for detecting the interaction between the anti-TS mAb 39 antibody and TS. Treatment with ethanolamine hydrochloride in DMSO first activated the cantilever´s tip, a simple and efficient protocol that allows the incorporation of nucleophilic NH_2_ handles to the tip for the conjugation of the PEG ligating chain [31,34]. Finally, the PEG1000-bis-VSO was “grafted-to” the aminofunctionalized tip surface by the click aza-Michael addition reaction of the VSO function to the NH_2_ groups by simple immersion of the tip into a basic solution of the reagent (Figure 1).

The unreacted VSO end of the resulting tip bounded to PEG1000-VSO was then used for the immobilization of the anti-TS mAb 39 antibody. For this purpose, two complementary strategies were implemented: random and oriented ligation (Figure 2). For the first case, ligation of the anti-TS mAb 39 antibody was attained by exploiting the click covalent tethering of the VSO groups to the nucleophilic endogenous amino acids of the antibody. Lysine residues are presumed to be the main target, considering that they are the most abundant surface amino acids of antibodies, although the conjugation to other less abundant aminated (arginine and histidine) or thiolated amino acids (cysteine) cannot be excluded as they are also suitable Michael donors in the reaction with VSO Michael acceptors. As the anchoring points are ubiquitous, the direct VSO-based conjugation of the anti-TS mAb39 to the tip bound to PEG-VSO should lead to a random ligation. To evaluate if this direct coupling alters the efficiency of the antibody-antigen binding, an oriented ligation approach was also undertaken. The strategy consisted of a two-step procedure based on the click-based VSO ligation of the tip to protein A, an affinity ligand that is used as a specific orientation crosslinker for its ulterior non-covalent binding to the Fc region of the anti-TS mAb 39 antibody. To preserve the integrity of the protein A/antibody complex, a final treatment with diethylene glycol bis-vinyl sulfonate (DEB-bis-VSO) was performed to obtain the click-based VSO covalent crosslinking of the two components of the protein A-antibody complex.

### 2.2. Isolation and Characterization of Ex-TcT and “TS-free” Ex-TcT 

**Figure 3:**
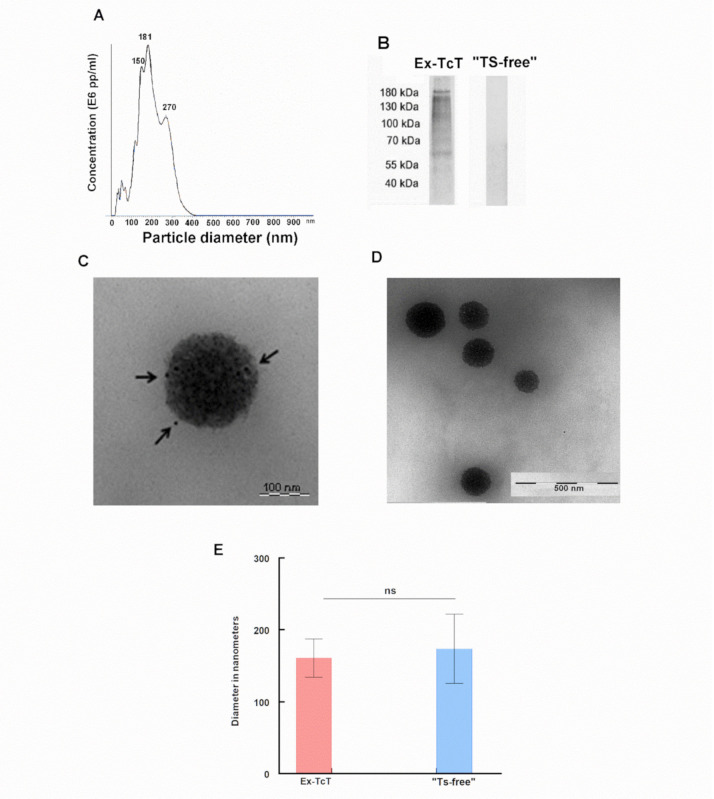
Characterization of Ex-TcT and “TS-free” Ex-TcT: (**A**) Nanoparticle tracking analysis size distribution of purified EVs from TcT of *T. cruzi*. (**B**) Western blot of Ex-TcT and “TS-free” Ex-TcT. All lanes were loaded with an equal amount of protein (30 µg). The lack of bands in EVs T indicates the loss of the corresponding epitope recognized by the anti-TS mAb 39 antibody. Molecular weight markers (precision plus protein all blue, Bio-Rad). (**C**) Ex-TcT of *T. cruzi*. Arrows highlight the gold particles of the labeled secondary antibodies that recognize TS. Scale bar: 100 nm. (**D**) “TS-free” Ex-TcT of *T. cruzi*. Gold particles are not observed as there is no recognition by the antibodies. Scale bar: 500 nm. (**E**) Means and SDs of the diameter measures obtained by TEM and performed using ImageJ (1.511j8p (NIH) software (http:\imgeJ.nih.gov/ij, accessed on 20 June 2022) and the Kolmogorov and Smirnov test. *p*-value: 0.5561. Not significant.

To demonstrate the ability of the anti-TS mAb 39 functionalized cantilever to sense *trans*-sialidases on the surface of EVs isolated from TcT of *T. cruzi,* the vesicles were obtained following a sequential differential centrifugation, filtration and ultracentrifugation process, a methodology previously reported by our research group [16]. Transmission electron microscopy (TEM) and nanoparticle tracking analysis (NTA) of the purified EVs confirmed the success of the methodology. Particularly, NTA and TEM results revealed a mean size of 143 ± 24 nm for most of the vesicles and a mode of 181 ± 24 nm (Figure 3A), suggesting that the isolated EVs so depending on the size they could correspond with exosomes released by the trypomastigote forms of the parasite (Ex-TcT).

In order to perform negative sensing experiments, a treatment of the Ex-TcT of *T. cruzi* with proteinase K was applied to eliminate TS and other proteins presents at the surface of EVs and obtain “TS-free” Ex-TcT. A comparative analysis by Western blot and TEM using immunogold labeling of both the native and proteinase K treated Ex-TcT reveal the success of the enzymatic treatment (Figure 3B–D). While the untreated Ex-TcT of *T. cruzi* show the characteristic TS pattern of high molecular weight proteins (187–67 kDa) in Western blot, these bands are practically absent in the “TS-free” Ex-TcT. Furthermore, immunogold labeling of the TS of the native Ex-TcT showed gold marks on the surfaces that were not present in the “TS-free” Ex-TcT.

### 2.3. Surface Analysis of Ex-TcT, “TS-free” Ex-TcT and Ex-TcT≈mAb 39 Immune Complexes

**Figure 4:**
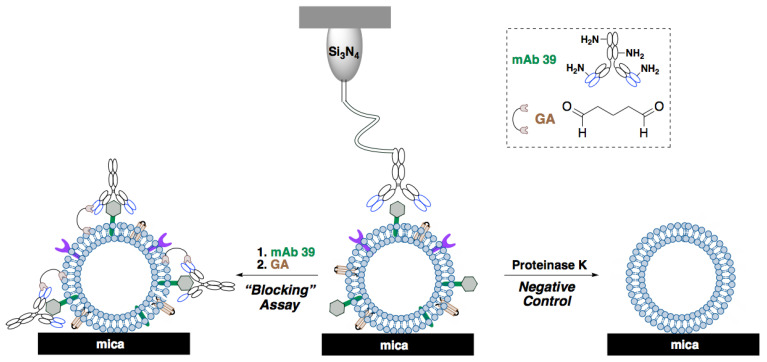
Immobilization of Ex-TcT, “TS-free” Ex-TcT (negative control), and Ex-TcT≈mAb 39 immune complexes (“blocking” assay) on mica sheets.

**Figure 5:**
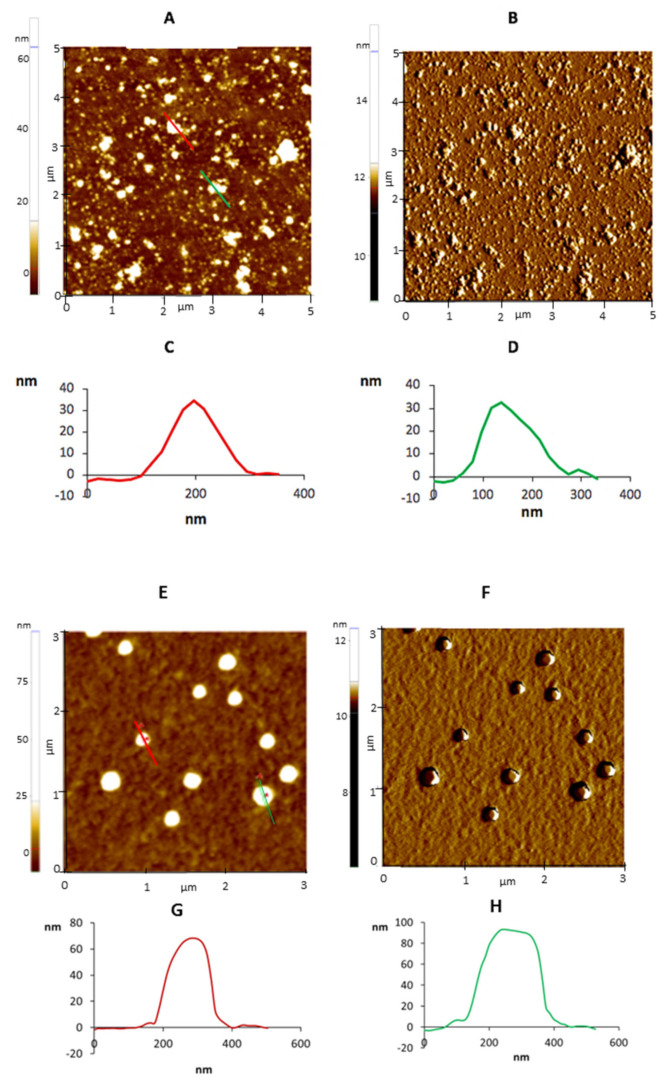
AFM images (5 × 5 µm) of Ex-TcT (**A**–**D**) Ex-TcT≈mAb 39 immune complexes (**E**–**H**) on mica: (**A**) Z-height; (**B**) amplitude signal; (**C**) line profile: 35 nm for height; (**D**) line profile: 32 nm for height; (**E**) Z-height; (**F**) amplitude signal; (**G**) line profile: 65 nm for height; (**H**) line profile: 87 nm for height.

AFM was employed to study the morphology and distribution of Ex-TcT and “TS-free” Ex-TcT through their non-covalent immobilization on mica sheets (Figure 4). Additionally, a further assay was designed through the incubation of the isolated Ex-TcT of *T. cruzi* with TS mAb 39 antibodies before their immobilization, for the formation of the corresponding Ex-TcT≈mAb 39 immune complexes. To ensure the stabilization of these complexes, their constituted elements were fixed by covalent crosslinking using glutaraldehyde. By the formation of the immune complex, TS on the surface of EVs are blocked, avoiding their recognition by the antibodies fixed to the cantilever. The results of Ex-TcT of *T. cruzi* confirmed the presence and integrity of the vesicles over the surface of the mica sheets. The imaging analysis revealed nanoparticles with a Z-height between 15 and 50 nm (Figure 5). The Z-height values obtained are in accordance with the expected dimensions for exosomes (>30 nm) [16,35,36,37]. For Ex-TcT≈mAb 39, Z-Height profile in the range of 60–90 nm is in accordance with the expected size obtained by adding the height of the Ex-TcT and the mAb 39. Furthermore, images showed single or clusters of EVs randomly distributed over the surface of mica with a proportion of vesicles and a distance between them required for the subsequent molecular recognition experiments.

### 2.4. Single-Molecule Recognition Imaging of Ex-TcT of T. cruzi

**Figure 6:**
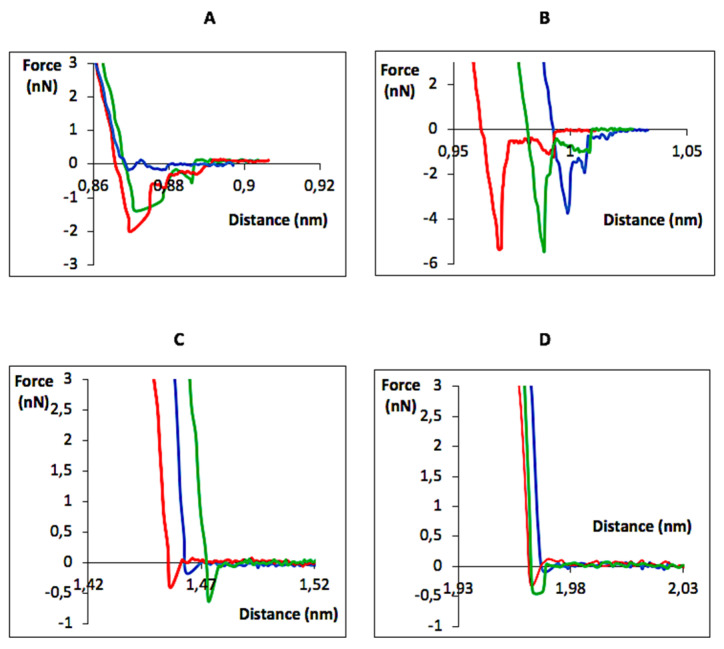
Single-molecule recognition experiments. Representative retraction force-distance curves between: (**A**) Oriented tip-bound anti-TS mAb 39 and Ex-TcT of *T. cruzi*; (**B**) random tip-bound anti-TS mAb 39 and Ex-TcT of *T. cruzi*; (**C**) random tip-bound anti-TS mAb 39 and “TS-free” Ex-TcT of *T. cruzi*; and (**D**) random tip-bound anti-TS mAb 39 and Ex-TcT≈mAb 39 immune complexes. Red, blue and green line are representing the overlay of different F-D patterns for the same interaction).

Breakdown forces between anti-TS mAb 39 antibodies and the Ex-TcT were measured to elucidate the work of adhesion and forces involved in the antigen-antibody recognition events (Figure 6). The binding was studied by applying a force to the receptor until the bond broke at a measurable unbinding force (nN-pN). The reliability of the measured unbinding forces was checked using numerous samples and repeating the measurements, leading to the generation of 65,536 curves. An exhaustive analysis evidenced the specificity of the measured unbinding forces for the Ex-TcT of *T. cruzi*, particularly in comparison to the negative and blocking control experiments (Figure 6C,D). In these last experiments, it was concluded that such unbinding forces do not exist after several hundred force curves were measured and recorded at different locations of the mica. Therefore, the EV-antibody interactions can be studied by the presence of TS anchored to the external part of the Ex-TcT membrane, which determines the existence of specific contact areas and, consequently, the existence of interactions between the Ex-TcT and antibodies. This result also suggests that the recognition of antigens by specific antibodies has a strong impact on the force curves, affecting the forces for the tip´s retraction. Moreover, in the case of native Ex-TcT, the different profiles obtained for the retraction force curves with respect to “TS-free” Ex-TcT (Figure 6A,B vs. Figure 6C) could be the result, at least in part, of a greater EV-antibody interaction due to the presence of numerous copies of the epitope recognized by the antibody. Finally, it is worth mentioning that the interaction pattern of the anti-TS mAb 39 with the Ex-TcT of *T. cruzi* for the oriented tip-bound experiment (Figure 6A) is slightly different respecting to the random-tip bound experiment (Figure 6B). Although the breaking force of the interaction is less intense in the first case, there is still an antibody-EV interaction. This observation can be tentatively assigned to a lower height for the EVs, which cause that the length of the linker is not adequate to give rise to a greater interaction.

**Figure 7:**
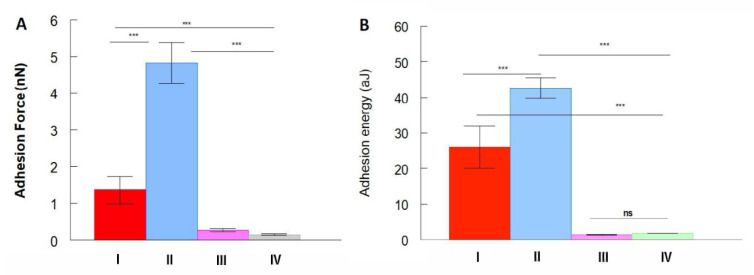
Mean values of (**A**) maximum adhesion force and (**B**) work of adhesion: (I) Oriented tip-bound anti-TS mAb 39 and Ex-TcT of *T. cruzi*. (II) Random tip-bound anti-TS mAb 39 and Ex-TcT of *T. cruzi*; (III) Random tip-bound anti-TS mAb 39 and “free-TS” Ex-TcT of *T. cruzi*; and (IV) Random tip-bound anti-TS mAb 39 and Ex-TcT≈mAb 39 immune complexes. The statistical test used was the Kruskal-Wallis test (non-parametric ANOVA) in combination with the GraphPad Instat statistical software (version 3.06). *** highly significant *p* < 0.001.

Figure 7 shows the maximum detachment force and work of adhesion obtained for all of the experiments. As expected, low values for work of adhesion were observed for “TS-free” Ex-TcT of *T. cruzi* and the fixed Ex-TcT≈mAb 39 immune complexes. However, a complete suppression of the adhesion to the antibodies was not observed in these control experiments. In contrast, the presence of TS in Ex-TcT of *T. cruzi* caused greater work of adhesion between the Ex-TcT and the tip, confirming that adhesion is mediated by the antigen-antibody recognition. Remarkably, a lower work of adhesion was observed when tips with an oriented functionalization were used with respect to the tips with a random ligation of the anti-TS mAb 39 antibodies. These surprising results were repeatable and, contrasted to most reports that employ protein A but have one antecedent in the literature [38]. The observation can be tentatively attributed to the increased elasticity of the protein A-mAb 39 complex that determine a longer carbon chain formed in this case by the linker plus the protein A bound to the antibody. The assembly of results support the recognition between the ligated anti-TS mAb 39 antibodies and the immobilized Ex-TcT of *T. cruzi*.

**Figure 8:**
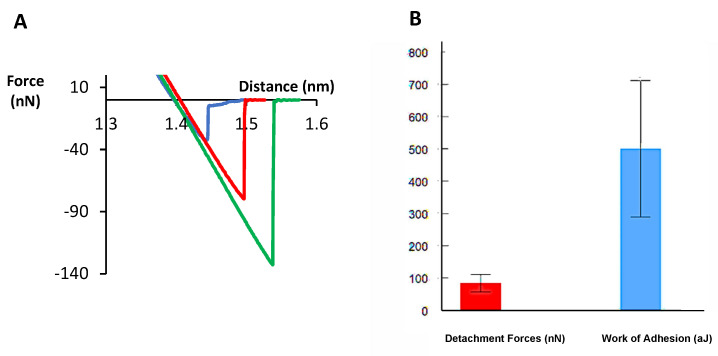
Molecular recognition force curves of the non-fixed Ex-TcT≈mAb 39 immune complexes. (**A**) Red, blue, and green lines represent three different patterns of interactions with the random tip-bound anti-TS mAb 39 and Ex-TcT≈mAb 39 immune complexes. (**B**) Mean values of maximum adhesion force and work of adhesion.

Force curves for the molecular recognition, as well as the maximum detachment force and the work of adhesion were also quantified in the case of the non-fixed Ex-TcT≈mAb 39 immune complexes (i.e., the immune complex not-treated with glutaraldehyde) to evaluate the influence of the crosslinking stabilization on the dynamic antigen-antibody complex. The results of the molecular interaction with the random tip-bound anti-TS mAb 39 show patterns differing to those obtained for the fixed Ex-TcT≈mAb 39 immune complexes (Figure 8).

**Figure 9:**
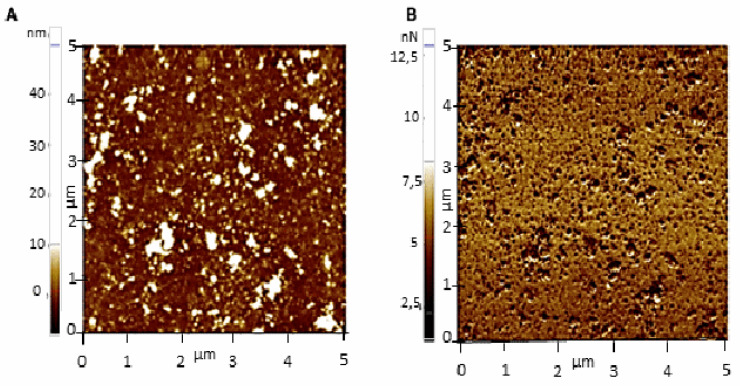
Topography and adhesion images of the molecular recognition of Ex-TcT of *T. cruzi* by anti-TS mAb 39 antibodies. (**A**) White dots represent the Ex-TcT distribution (20–30 nm for height and 50–330 nm in diameter). (**B**) Black dots represent the recognition of TS epitopes by the anti-TS mAb 39 antibodies during the scan process of the surface. Images were simultaneously acquired (5 × 5 µm).

The breaking forces of specific intermolecular interactions are used in these experiments as a contrast parameter to create the adhesion (interaction) images. In addition, the adhesion force between the tip and the surface, as well as the contrast, allow the substrate to be mapped with each of the different biomolecules deposited in these experiments. Figure 9 shows the topographic map and the corresponding adhesion image for the molecular recognition process between the functionalized tip on the surface of the Ex-TcT of *T. cruzi*. The Ex-TcT deposited on the mica are observed in the Z-Height image as granules with the color corresponding to their relative height (Figure 9A), while black spots show the recognition of the TS epitopes by the anti-TS mAb 39 antibodies (Figure 9B). The contrast that is usually induced in the adhesion image can be obscured by variation of the contact area between the tip and the sample in the molecular recognition experiment, either because of the speed of the interaction or the size of the deposited Ex-TcT [39]. Additionally, the contrast in the adhesion image allows to visualize the position of the interaction domains in the Ex-TcT.

## 3. Discussion

The goal of this work was the detection and localization of functional TS molecules at the surface of the Ex-TcT of *T. cruzi* by employing AFM-based single-molecule recognition force microscopy and spectroscopy. These techniques require the tip functionalization with a sensor molecule, the anti-TS mAb 39 antibodies, for the specific binding to the cognate TS target sites of the Ex-TcT-coated surface. Our results demonstrate that the covalent binding of the anti-TS mAb 39 antibodies to the tip is an adequate strategy over alternative non-covalent and non-specific approaches [29] because it ensures that the linkage between the tip and the anti-TS mAb 39 antibodies is much higher than the rupture force of the antibody–antigen complex [31].

To achieve the covalent attachment, the use of a long PEG1000 chain acting as a flexible tether has been demonstrated to be adequate. This is a robust and advantageous methodology that facilities the reorientation of the sensor molecule linked to the tip, and the specific binding and discrimination from non-specific tip-surface interactions. The benefits of the flexibility of PEG cross-linkers have been particularly validated in reported antibody–antigen recognition studies [33]. The PEG1000 cross-linker promotes specific unconstrained receptor–ligand recognition by providing translational and rotational degrees of freedom [40]. In this way, the anti-TS mAb 39 antibodies can freely orient and diffuse within a certain volume.

In the current state of the art, a versatile toolbox of reactive PEG linkers and linking protocols are available for the functionalization of silicon and silicon-nitride tips with virtually any probe molecule [29,30,31]. The most common strategy relies on the aminofunctionalization on the chemically inert tip surface, followed by the amine-based conjugation of a reactive bifunctional PEG-crosslinker and ulterior coupling of the unreacted end of the tether of the sensor molecule. A panoply of PEG homo- and heterocrosslinkers containing, respectively, equal or different reactive groups at each terminus of the PEG chain are currently available [30]. In most AFM applications, heterobifunctional PEG crosslinkers are preferred for the tip functionalization to avoid undesirable crosslinking of adjacent amine groups on the activated tip surface or loop formation, side reactions that are plausible when using homobifunctional PEG reagents [31]. Despite the possibilities that the presence of two different reactive functions offers for the linking of two different functional targets and the control of the kinetics of the conjugation reactions at both termini of the linker, the necessity of multistep preparative procedures, including protection and deprotection reactions, with the concomitant increase in operational complexity and costs, are important drawbacks associated to heterocrosslinkers. 

In this study, the maximization of the operational simplicity and the efficiency of the tip-antibody tethering are key requirements. To these ends, and regarding the tip activation with amines, the selection of the “ethanolamine method” demonstrated to fulfill those requirements [33]. The selection of this protocol over the traditional aminosilanization methodology was based on its documented simplicity and efficiency in measuring the unbinding forces that our results have corroborated [31,34]. The ethanol amine hydrochloride treatment allows the formation of an aminoalkane monolayer on the silicon tip surface with a low surface density through an etherification reaction, forming Si-O-C bonds in a simple and efficient manner.

Respecting the antibody-tip conjugation, the homobifunctional PEG1000-bis-VSO reagent reported here has proved to be highly efficient for the straightforward and simple attachment of both the aminofunctionalized tip and the anti-TS mAb 39 antibodies. Nowadays, the N-hydroxisuccinimide (NHS) ester is the prevalent reactive group in most reported PEG linkers used in AFM for the coupling to amine-functionalized tips and surfaces or to amine-containing probes [30,31]. The activation of carboxylated PEG derivatives through NHS esters is used not only for the access to the NHS-PEG-NHS homobifunctional crosslinker but also for the preparation of most heterobifunctional PEG crosslinkers that incorporate others reactive groups for their implementation in diverse conjugation strategies. However, the NHS ester is prone to rapid hydrolysis with increasing pH. As the amine group requires basic conditions for its deprotonatation to increase nucleophilic activity, handling the NHS ester reaction in aqueous solutions is difficult and a fine-tune of the pH and reaction time are required to achieve optimal outcomes [41]. In contrast, the reactive VSO groups incorporated into PEG1000-bis-VSO offer attractive features. Reagents containing the vinyl sulfonyl motif (vinyl sulfones -VS- and vinyl sulfonates -VSO-) are Michael acceptors that enable the 1,4-conjugated addition of nucleophilic Michael donors [32,42]. This reaction is included in the core group of highly efficient click reactions [43]. Bioconjugation techniques based on vinyl sulfones have found wide applications because their click reactions with endogenous nucleophilic amino acids (cysteine, lysine and histidine) benefit from mild reaction conditions in aqueous media, high conversions and favorable reaction rates leading to stable β-heterosubstituted sulfone adducts [44,45]. Additionally, the methodology offers the possibility of a good control for the chemoselective ligation to cysteine over other aminated amino acids by using a well-tuned pH. In fact, the selective coupling of thiols has been exploited for attaching probe molecules to AFM tips through the VS-containing PEG heterocrosslinkers VS-PEG-NHS and VS-PEG-N_3_. These heterocrosslinkers shown to be robust reagents for the irreversible site-specific ligation of SH-containing probes in few examples in the literature [40,41,46,47]. 

On the basis of our expertise on click chemistry in bioconjugation [48,49], the VSO group was purposely selected in this study over the vs. function for the preparation of the homo-crosslinker PEG1000-bis-VSO to attain the mentioned simplicity-efficiency tandem of our linking protocol. The vinyl sulfonate derivatization of PEG1000 is an easier and less demanding reaction compared to the vinyl sulfone functionalization [32]. Alternatively, the VSO group is a suitable Michael acceptor for the click aza-Michael addition of the NH_2_ groups of both the tip surface and the most abundant surface-exposed lysine residues of proteins or antibodies. By this way, the pre-derivatization or the engineering of the probe, cost- and time-consuming steps required in most of the site-specific reported protocols is avoided by taking advantage of the native nucleophilic group of endogenous residues of lysine despite the non-specific ligation attained by our VSO-based protocol bioconjugation. 

Notwithstanding that the direct VSO-based conjugation of the anti-TS mAb 39 antibodies to the tip-bound PEG-VSO benefits from the existence of multiple points of attachment, a distortion of the anti-TS mAb 39 receptors and detriment of the ulterior antibody-antigen binding cannot be excluded in this random ligation strategy. For this reason, the aforementioned oriented ligation of the anti-TS mAb 39 antibodies to the tip mediated by protein A was also undertaken in view of preserving the antibody binding region. Protein A, a naturally occurring surface protein from *Staphylococcus aureus* that exhibits antibody binding domains with affinity mainly to the fragment crystallizable (Fc) part of IgG, is an affinity ligand that has been previously used as an orientation crosslinker for the non-covalent ligation of antibodies to AFM tips [38,50,51,52]. In this study, the immobilization of protein A on the tip-bound PEG-VSO was expected to produce a control of the molecular orientation of the anti-TS 39 mAb antibodies since the Fab domains should be free after attachment to the AFM cantilever´s surface. The non-covalent binding of the anti-TS mAb 39 antibodies to protein A was easily performed by simply immersion of the tip functionalized with protein A, thus creating the tip-PEG-protein A/mAb 39 sensor. Moreover, the click VSO chemistry was also exploited for performing the covalent crosslinking of protein A with the anti-TS mAb 39 antibodies by using the short linker diethylene glycol bis-vinyl sulfonate to prevent the complex Protein A-IgG from decomposing and entering into equilibrium, releasing and trapping the Fc parts of new IgGs. 

Results show that all of the click VSO-based ligations aimed at the functionalization of the cantilevers are experimentally simple and effective, regardless of where they are applied: tethering of the PEG linker to the aminofunctionalized tip, random ligation of protein A or the anti-TS mAb 39 antibodies to the functionalized tip, and cross-linking of the diethylene glycol bis-vinyl sulfonate between the elements of non-covalent systems (protein A/anti-TS mAb 39 antibodies and Ex-TcT≈mAb 39 immune complexes). 

As commented, given the high specificity of the anti-TS mAb 39 antibodies for TS of Ex-TcT of *T. cruzi*, the AFM study of this antibody-antigen interaction was proposed in terms of the breaking forces for the resulting antibody-EV complex, an approach previously reported [28,53,54,55,56]. Data analysis of each force-distance curve and the resulting graphs representing the values of adhesion force and adhesion work after the tip-sample interaction reveals the specificity of the force spectroscopy measurements (Figure 6). This is clearly proven in the negative and blocking experiments performed with the “TS-free” Ex-TcT of *T. cruzi*, and Ex-TcT≈mAb 39 immune complexes, respectively. Figure 6C shows a considerable decrease in the forces obtained for “TS-free” Ex-TcT, while Figure 6D demonstrates that, since the retraction force curves are almost null, there is no recognition of the epitopes of the TS of the Ex-TcT because of the blocking by the Fab fragment of the anti-TS mAb 39 antibodies in the Ex-TcT≈mAb 39 immune complex. However, the elimination of surface proteins in the “TS-free” Ex-TcT did not completely suppress the adhesion to the antibodies, indicating that some non-specific interactions still occur. The observation points to the existence of interactions of electrical charges or hydrophobic interactions between the “TS-free” Ex-TcT and the functionalized tip, and/or between the membrane of the Ex-TcT and the hydrophobic parts of the anti-TS mAb 39 antibodies. 

Regarding the functionalization of the tip by the oriented ligation of the anti-TS mAb 39 antibodies mediated by protein A, the data show that the approach also led to specific interactions. However, and regarding the crosslinking between protein A and the antibody to prevent the complex protein A-IgG from decomposing, Figure 6A shows that, in this case, the retraction forces display less defined curves, with a higher number of fluctuations compared to the curves obtained using the random functionalized cantilevers (Figure 6B). These results can be tentatively assigned to the greater elasticity of the protein A-mAb 39 antibody complex. Moreover, the random ligation of the antibody shows the greatest work of adhesion and detachment forces. Marked peaks are observed at 5.3, 5.4, and 3.7 nN forces (red, green and blue curves, respectively) corresponding to rupture events at a higher force value than for the oriented-tip bound antibody in Figure 6A (2.00, 1.62 and 0.181 nN; red, green and blue curves, respectively). The measurement of intramolecular forces implies some requirements on the stability of the coupling between molecules, and on the tip and surface itself. Furthermore, for measuring intermolecular forces between the tip and the molecule deposited on the surface, the breaking forces are always greater than that corresponding to the intramolecular domains. On this basis, the comparisons between results of this study and similar studies [33,53] show that the values are consistent with the reported for antigen-antibody bonds, which exhibit forces up to several nanonewtons because this force is high enough to break several antibody-antigen bonds at the same time [33,57]. Furthermore, “TS-free” Ex-TcT showed, as expected, significantly lower adhesion and detachment forces as the IgGs did not bind to the epitopes of the TS antigens (Figure 6C). 

Regarding the maximum detachment forces (Figure 7A) and the work of adhesion extracted from the adhesion force areas (Figure 7B), the obtained values for both parameters are in good agreement. As expected, the maximum work of adhesion values are observed for the Ex-TcT of *T. cruzi* with the anti-TS mAb 39 antibodies random tip-bound (42.68 aJ, II) and the oriented tip-bound (26.92 aJ, I), while very low values are obtained by the random ligation approach in the control experiments with the”TS-free” Ex-TcT (1.44 aJ, III) and the Ex-TcT≈mAb 39 immune complexes (1.83 aJ, III). These observations are in concordance to similar reports found in the literature [58,59]. Moreover, the low values found in the control experiments justify the relationship between the association and dissociation of the Fabs from the antibodies and the recognized epitopes of the antigen, which resembles a dynamic equilibrium process as occurs in solid-phase immunoassays [60]. At this point, it is important to highlight that the rapid antigen-antibody binding process cannot be completely hindered when the antibodies do not fully occupy the surface of the Ex-TcT of the immune complexes. In the case of the non-fixed Ex-TcT≈mAb 39 immune complexes, results found show values in a wide range of forces of interaction and work of adhesion (39–133 nN and 113–842 aJ, respectively) (Figure 8). These high differences are typical of dynamic interactions that change according to the contact area between the interacting molecules: sometimes the antibody mAb 39 tethered to the AFM tip interacts on the surface of the Ex-TcT not totally covered by antibodies, while in other times on the antibody itself. Consequently, the dynamic of the antigen-antibody interactions supports the need for fixation of the Ex-TcT≈mAb 39 immune complex.

Finally, the AFM topography and molecular recognition images obtained simultaneously (Figure 9) further demonstrate that the membranes of the EVs secreted by TcT of *T. cruzi* are enriched in TS. The mapping of the binding sites performed in the native EVs, “TS-free” EVs and the EV≈mAb 39 immune complexes could help to understand the EV-cell interactions, not only in this model using EVs of *T. cruzi*, but also in other EVs of diagnostic interest.

## 4. Materials and Methods

### 4.1. Multi-Step Conjugation of Anti-TS mAb 39 Antibodies to the Cantilever

#### 4.1.1. Aminofunctionalization

The functionalization of the cantilever was performed by the ethanolamine method [33]. Briefly, ethanolamine hydrochloride (1.65 g) was dissolved in DMSO (5 mL) by gentle heating at 70 °C, and, subsequently, 10% (*v*/*v*) of molecular sieves (4 Å) were added. The solution was allowed to cool to come at room temperature. Commercial silicon nitride AFM tips (NSC-36 C), with a spring constant of 0.6 N/m and purchased from Park Systems, were then introduced into the DMSO solution and kept under vacuum at room temperature for one week. Subsequently, the cantilevers were washed twice by immersion in DMSO for a few minutes and dried with a gentle stream of nitrogen. When the tips were not used immediately, they were stored in a desiccator under an argon atmosphere.

#### 4.1.2. Preparation of Tip-Bound PEG-VSO

The VSO functionalization of the amino-activated cantilever was performed by covalent coupling to PEG1000-bis-VSO. Briefly, homobifunctional PEG-bis-VSO (130 mg) and triethylamine (100 µL) were dissolved in DMSO (5 mL). The tips were then immersed in the prepared solution overnight. Finally, each tip was washed individually by subsequent immersion in DMSO and dichloromethane for 5 min. After the washing step, the tips-bound PEG-VSO were air dried and stored in a desiccator under an argon atmosphere.

#### 4.1.3. Ligation of Anti-TS mAb 39 Antibodies

The ligation of the antibodies was performed by two alternative methodologies: 

*(a) Random ligation.* The cantilevers with the tip-bound PEG-VSO were immersed in a solution of the anti-*trans*-sialidase mAb 39 antibodies (1:100) in carbonate buffer (0.1 M), pH 8.3, for 2–4 h for the direct and random ligation of the antibody to the PEG appendages.

*(b) Oriented ligation.* The cantilever with the tip-bound PEG-VSO was first incubated with 100 µg protein A (Sigma-P6031) dissolved in 0.22 µm of the previously filtered PBS buffer (0.125 M), pH 7.2, for 1 h. The remaining reactive vinyl sulfonate groups of the unreacted PEG linkers were blocked by treatment with glycine (0.2 M) for 1 h at room temperature. Then, the tips were immersed in a solution of the anti-*trans*-sialidase mAb 39 antibodies (1:100) in carbonate buffer (0.1 M), pH 8.3, for 2–4 h to bind the antibody to the protein A through the Fc region. To prevent decomposition of the complex and release of the antibody, a lateral crosslinking of protein A to the antibody was carried out by treatment of the tips with diethylene glycol bis-vinyl sulfonate (20% in diethylene glycol) for 2 h. The remaining free VSO groups of unreacted cross linkers were blocked by treatment with 0.2 M glycine in carbonate buffer as described above. 

### 4.2. Cell Culture, Parasite Strain and Culture of Trypomastigotes of T. cruzi

The cell culture, the infection process and methodology for harvesting trypomastigotes obtained from cells culture, for the subsequent isolation of EVs, was performed as previously described [16]. Briefly, Vero cells (ECACC 84113001) were cultured in Nunc cell culture flasks of 75 cm^2^ surface (Thermo Fischer Scientific, Waltham, MA, USA) in Minimal Essential Medium (MEM) (Sigma Aldrich, St. Louis, MO, USA), supplemented with 10% fetal heat inactivate bovine serum (Gibco, Waltham, MA, USA) (iFBS), and antibiotics (penicillin 100 U/mL, streptomycin 100 g/mL). Cell cultures were maintained at 37 °C in a humid atmosphere enriched with 5% CO_2_. TcT of *T. cruzi* were obtained after intracellular multiplication of the parasite (96 h) within cells infected with *T. cruzi* Pan4 trypomastigotes (TcIa + TcId) following a protocol previously described [61] 

### 4.3. Isolation of Ex-TcT of T. cruzi

The purification of Ex-TcT of *T. cruzi.* was performed following a methodology previously described [62]. For this purpose, 5 × 10^7^ TcT collected from the supernatant of infected cell cultures were washed (4 times) by centrifugation (1500× *g* for 15 min) in ultrafiltered PBS (0.22 µm) and incubated for 5 h in 5 mL of RPMI1640 medium (Sigma Aldrich, St. Louis, MO, USA) supplemented with 10% exosome-free iFBS. The viability of trypomastigotes after the EV secretion was assessed by the trypan blue exclusion assay, maintaining 99% viability after the incubation time. Purification of EVs was carried out by a differential centrifugation (3500× *g* for 15 min; the supernatant was centrifuged at 17,000× *g* for 20 min; collection of the supernatant and filtration through 0.22 µm and finally centrifugation of the filtrate at 100,000× *g* for 18 h). The resulting pellet was washed three times by ultracentrifugation in sterile filtered PBS. The isolation and purification of Ex-TcT was evaluated by transmission electron microscopy and by nanoparticle tracking analysis, as described in a previous work [16]. 

### 4.4. Enzymatic Treatment of Ex-TcT of T. cruzi. for the Production of “TS-free” Ex-TcT 

Samples of Ex-TcT of *T. cruzi* (40 µg of total proteins) were treated with proteinase K (final concentration 0.5 mg/mL) (Sigma-Aldrich, St. Louis, MO, USA) for 30 min at 37 °C, as previously described [62]. After the treatment, the samples were washed twice in sterile Hanh’s solution by ultracentrifugation (120,000× *g* for 4 h) to remove proteinase K. The “TS-free” Ex-TcT pellets were finally resuspended in a solution of sterile PBS plus an EDTA-free protease inhibitor cocktail (Roche, Basel, Switzerland) filtered through 0.22 µm filters. 

### 4.5. Electrophoretic Separation of Proteins from EVs from TcT of T. cruzi and Western Blot 

Proteins from Ex-TcT of *T. cruzi* were precipitated overnight in 4 volumes of acetone at −20 °C. The samples were centrifuged at 13,000× *g* for 10 min at 4 °C and two washing steps with cold acetone were performed. The acetone was evaporated under a nitrogen stream and the precipitated proteins were quantified using the Micro-BCA protein assay kit (Thermo Fischer Scientific, Waltham, MA, USA), electrophoresed on 12% SDS-PAGE gels and transferred to PVDF membranes (Bio-Rad, Alcobendas, Madrid) in a Turbo Trans-Blot transfer system (Bio-Rad, Alcobendas, Madrid). The transferred membranes were immersed in blocking buffer (PBS, 0.1% Tween 20, and 4% nonfat dry milk) and incubated under shaking at 4 °C for 2 h. The blocked membranes were incubated with a dilution of anti-TS mAb 39 antibodies (1:1000) overnight, at 4 °C. After this time, the membranes were washed and then incubated for 1 h with peroxidase-conjugated goat anti-mouse IgGs (1:1000) (Dako Agilent Pathology Solutions, USA). The positive reaction was visualized using the Clarity ECL Western substrate (BioRad, Spain) in a ChemiDoc Imaging system (BioRad, Spain).

### 4.6. Transmission Electron Microscopy and Immunogold Labeling

For visualization under TEM, the purified Ex-TcT of *T. cruzi* were resuspended in 30 µL Tris-HCl (pH 7.3) and 5 µL of the suspension was adsorbed directly onto Formvar/carbon coated grids. After 30 min, the grids were washed in PBS, fixed in 1% glutaraldehyde for 30 min, washed again in PBS, and stained and contrasted with 2% (*v*/*v*) uranyl acetate. Finally, the samples were observed under a Carl Zeiss SMT LIBRA 120 PLUS TEM microscope. The size of the nanoparticles was measured using the Image J 1.41 software.

The purified Ex-TcT and ”TS-free” Ex-TcT of *T. cruzi* were analyzed by immunogold labeling for detecting surface TS using the anti-TS mAb 39 antibodies. In this case, the EV suspensions were adsorbed on the grids and fixed in glutaraldehyde. To block the aldehyde groups, the grids were treated in 0.02 M glycine (pH 7.3) for 30 min and then in ultrafiltered blocking buffer (0.05% Tween 20 in PBS, pH 7.4, plus 1% non-fat milk) for an additional period of 30 min. Finally, the grids were incubated with a dilution of the anti-TS mAb 39 antibodies (1:50) for 2 h, washed and incubated again with a goat anti-mouse IgG antibody (whole molecule) labeled with gold (20 nm) (Sigma-Aldrich, St. Louis, MO, USA) for 2 h. The final contrast was performed using 2% (*v*/*v*) uranyl acetate and the samples were analyzed on a Carl Zeiss SMT LIBRA 120 PLUS TEM microscope.

### 4.7. Generation of Ex-TcT≈mAb 39 Immune Complexes

A mixture of the isolated Ex-TcT of *T. cruzi* (100 µL of a 0.2 µg/µL sample) and the anti-TS antibody mAb 39 (200 µL, protein concentration of 0.84 µg, 1:1000 dilution) was incubated with orbital shaking (10 revolutions/min) at 37 °C for 1 h. After this time, three subsequent washes with PBS with protease inhibitors (Roche, Switzerland) were performed at 4 °C for 2 h at 120,000× *g* (Beckman Coulter, Brea, CA, USA). The final pellets were resuspended in 200 µL of sterile-filtered PBS and maintained at 4 °C until use of the resulting Ex-TcT≈mAb 39 immune complexes. To prevent the dissociation of this complexes, they were fixed to attain their stabilization by treatment with 2.5% glutaraldehyde (crosslinked) in carbonate buffer at pH 8.2 for 2 h, followed by ultracentrifugation as described above and the subsequent incubation of the pellets with 1 M glycine (pH 7.3) for 30 min to block aldehyde groups. The fixed Ex-TcT≈mAb 39 immune complexes thus obtained were finally washed 3 times by ultracentrifugation prior to their use in the interaction studies.

### 4.8. Atomic Force Microscopy

#### 4.8.1. Adsorption of Samples to Mica Sheets

Samples of the Ex-TcT, “TS-free” Ex-TcT and Ex-TcT≈mAb 39 immune complexes (10 µL) were deposited onto freshly cleaved mica muscovite. After 10 min, three washing steps were carried out with 50 µL of MilliQ water (Millipore, Burlington, MA, USA) to remove salts and loosely bound EVs and immune complexes. The samples were dried with a gentle stream of argon before imaging.

#### 4.8.2. Topographic Measurements and Imaging

Non-contact mode AFM imaging was performed (Atomic Force Microscopy Unit of the “Centro de Instrumentación Científica”, University of Granada, Spain) using an NX-20 instrument (Park Systems, Suwon, Korea) and ACTA cantilevers (K = 40 N m^−1^ and *f* = 320 kHz). Images were typically acquired as 256 × 256 pixels at a scan rate of 0.5–0.7 Hz. Subsequently, images were processed and analyzed using XEI software (Park Systems, Suwon, Korea). Representative images of the samples were obtained by scanning at least 3 different locations on at least 3 different samples of the same nature.

#### 4.8.3. Force Spectroscopy

The interaction between the AFM functionalized tip and the sample deposited in mica was measured by moving the tip in a perpendicular direction to the surface while measuring the force on the tip. For a brief explanation, force-Z Height curves start from force = 0, when the tip approaches to the sample with an increasing force until the tip and sample come into contact. The push of the tip toward the surface requires high forces that, in the end, produce the bending of the cantilever. The retraction of the tip produces a peak in the curve, indicating that the separation between the tip and the sample has occurred and the bond between the two interacting molecules has been broken. The specific length of the peak matches the length of the PEG connector (approximately 20 nm) when fully stretched, indicating that the strength can be attributed to a specific event.

The molecular recognition of TS at the surface of Ex-TcT of *T. cruzi*, the interaction between the functionalized tip and the surface of the Ex-TcT was evaluated by analyzing the force (nN)-distance(nm) curves obtained for each pixel in the scanned images (256 × 256 pixels). “TS-free” Ex-TcT and Ex-TcT≈mAb 39 immune complexes were included as negative controls. The PinPoint^TM^ mode developed by Park Systems was employed. The technique avoids frictions, artifacts and noise, facilitating the acquisition of reproducible and reliable topography and maps. The measurement procedure comprises three steps: first, the XY scanner stops during acquisition; second, the tip approaches the surface, measures mechanical properties and retracts from the surface over 4 ms to achieve an interaction force preset (15 nN); and, third, records the approach height and maintain the Z distance. Then, the tip is retracted and moved to the next pixel (Figure S1).

## 5. Conclusions

To summarize, an antibody-tethered AFM probe is presented here as a pivotal element for the detection and localization of TS molecules at the surface of Ex-TcT of *T. cruzi*, taking advantage of the specific recognition of TS by the anti-TS monoclonal antibody (mAb 39). The ad hoc mAb 39 covalent tip functionalization, performed by exploiting the vinyl sulfonate click-chemistry, demonstrated to be a reliable method for the tip-antibody tethering with outstanding characteristics such as simplicity and efficiency. The AFM-based single-molecule recognition force microscopy and spectroscopic data obtained with the functionalized tip demonstrate that the membranes of the EVs of trypomastigotes are enriched in TS and a mapping of their TS binding sites with submicrometer-scale resolution is provided. The finding reported may help to understand the Ex-TcT membrane from its glycobiological perspective and to gain insights into the glycointeractions of this subpopulation of the secretome of *T. cruzi* with the mammalian host cells. Moreover, the vinyl sulfonate-based functionalization of the tips is an open strategy that can be implemented for the ligation of other sensing molecules with applicability in diverse AFM scenarios.

## Supplementary Materials

The following supporting information can be downloaded at: https://www.mdpi.com/article/10.3390/ijms23137193/s1.
