# Supplementary material for: Molecular Recognition of Surface Trans-Sialidases in Extracellular Vesicles of the Parasite Trypanosoma cruzi Using Atomic Force Microscopy (AFM)
Source: Int J Mol Sci. 2022 Jun 28;23(13):7193. doi: 10.3390/ijms23137193 (PMC9266976; doi:10.3390/ijms23137193)
Supplement: Supplementary file 1 [file ijms-23-07193-s001.zip › ijms-1774162-supplementary.pdf]

SUPLEMENTARY MATERIALS

AFM molecular recognition of surface *trans*-sialidases of exosomes from *Trypanosoma cruzi*

Alexa Prescilla-Ledezma[1,5 *], Fátima Linares [2], Mariano Ortega-Muñoz [2], Lissette Retana Moreira[1,6,7], Ana Belén Jódar-Reyes [4], Fernando Hernandez-Mateo[3], Francisco Santoyo-Gonzalez[3] and Antonio Osuna[1]

[1] Grupo de Bioquímica y Parasitología Molecular (CTS 183), Departamento de Parasitología, Campus de Fuentenueva, Instituto de Biotecnología, Universidad de Granada, 18071 Granada, Spain

[2] Unidad de Microscopía de Fuerza Atómica, Centro de Instrumentación Científica, Universidad de Granada, 18071, Granada, Spain

[3] Departamento de Química Orgánica, Facultad de Ciencias, Universidad de Granada, 18071 Granada, Spain

[4] Grupo de Fisica de Fluidos y Biocoloides (FQM 115), Excellence Research Unit Modeling Nature (MNat), Departamento de Física Aplicada, Facultad de Ciencias, Universidad de Granada, 18071 Granada, Spain

[5] Departamento de Microbiología Humana, Facultad de Medicina, Universidad de Panamá, Ciudad de Panamá 0824, Panamá.

[6] Departamento de Parasitología, Facultad de Microbiología, Universidad de Costa Rica, San José 2060, Costa Rica.

[7] Centro de Investigación en Enfermedades Tropicales (CIET), Universidad de Costa Rica. San José 2060, Costa Rica

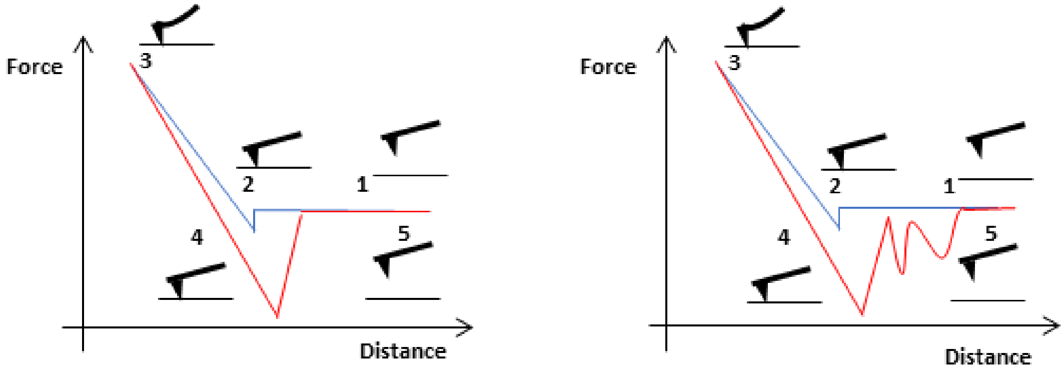

Figure S1: Schematic representation of examples of F-D plots for the tip-sample interaction (left) and for a molecular recognition experiment (right).
